# Supplementary material for: Helicobacter pylori infection and α-synuclein pathology drive parallel neurodegenerative pathways in the substantia nigra
Source: J Neuroinflammation. 2025 Dec 22;22:293. doi: 10.1186/s12974-025-03596-z (PMC12723842; doi:10.1186/s12974-025-03596-z)
Supplement: Supplementary file 1 — Supplementary Material 1. [file 12974_2025_3596_MOESM1_ESM.docx]

**Supplementary Materials for:**

***Helicobacter pylori* infection and α-synuclein pathology drive parallel neurodegenerative pathways in the substantia nigra**

Alejandro Soto-Avellaneda^1^, Alice Prigent^1^, Lindsay Meyerdirk^1^, Noah Schautz^1^, John Andrew Pospisilik^2^, Lena Brundin^1^, Michael X. Henderson^1^

^1^Department of Neurodegenerative Science, Van Andel Institute, Grand Rapids, MI 49503

^2^Department of Epigenetics, Van Andel Institute, Grand Rapids, MI 49503

**
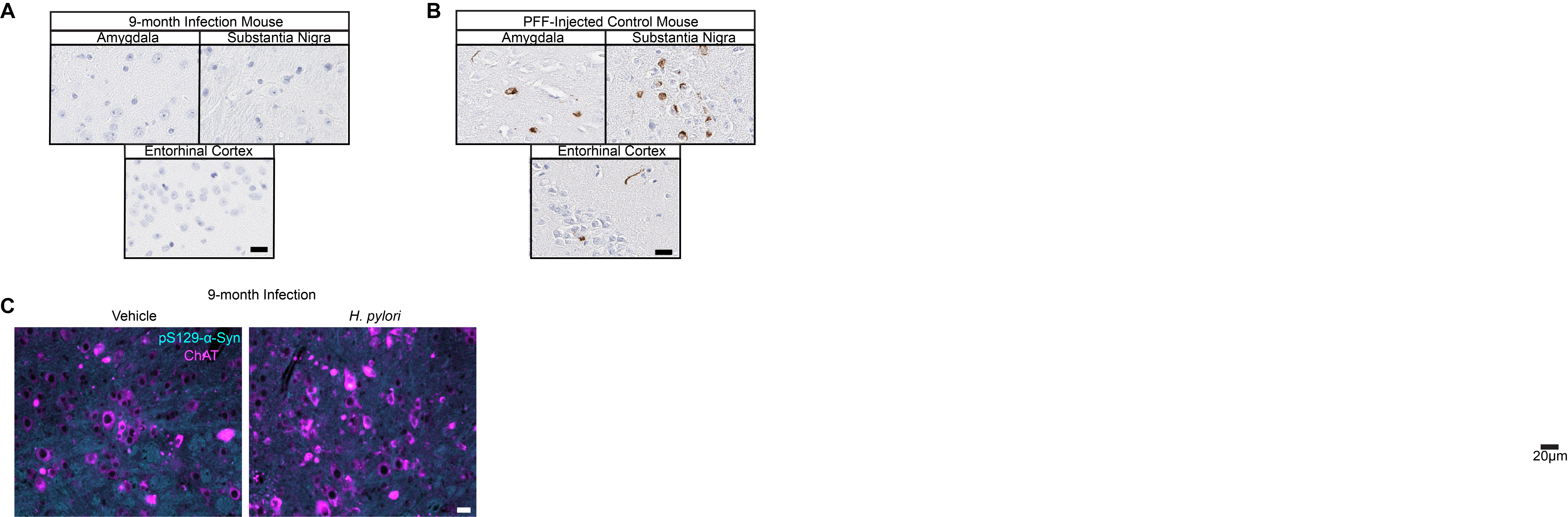
**

**Fig. S1. Aged *H. pylori*-infected mice do not show signs of α-synucleinopathy in the brain.** **A.** representative images of *H. pylori*-infected 9-month infection amygdala, substantia nigra, and entorhinal cortex stained for pS129 α-synuclein. **B.** Representative images of α-synuclein PFF-injected positive control amygdala, substantia nigra, and entorhinal cortex stained for pS129 α-synuclein. **C.** Representative image of vehicle-treated and *H. pylori-*infected hindbrains showing the dorsal motor nucleus of the vagus nerve (DMX) stained for choline acetyltransferase (ChAT) and pS129 α-synuclein. The pS129 α-synuclein channel intensity has been increased to demonstrate that the channel was imaged, but only background staining is visible. Scale bars = 20 µm.

**
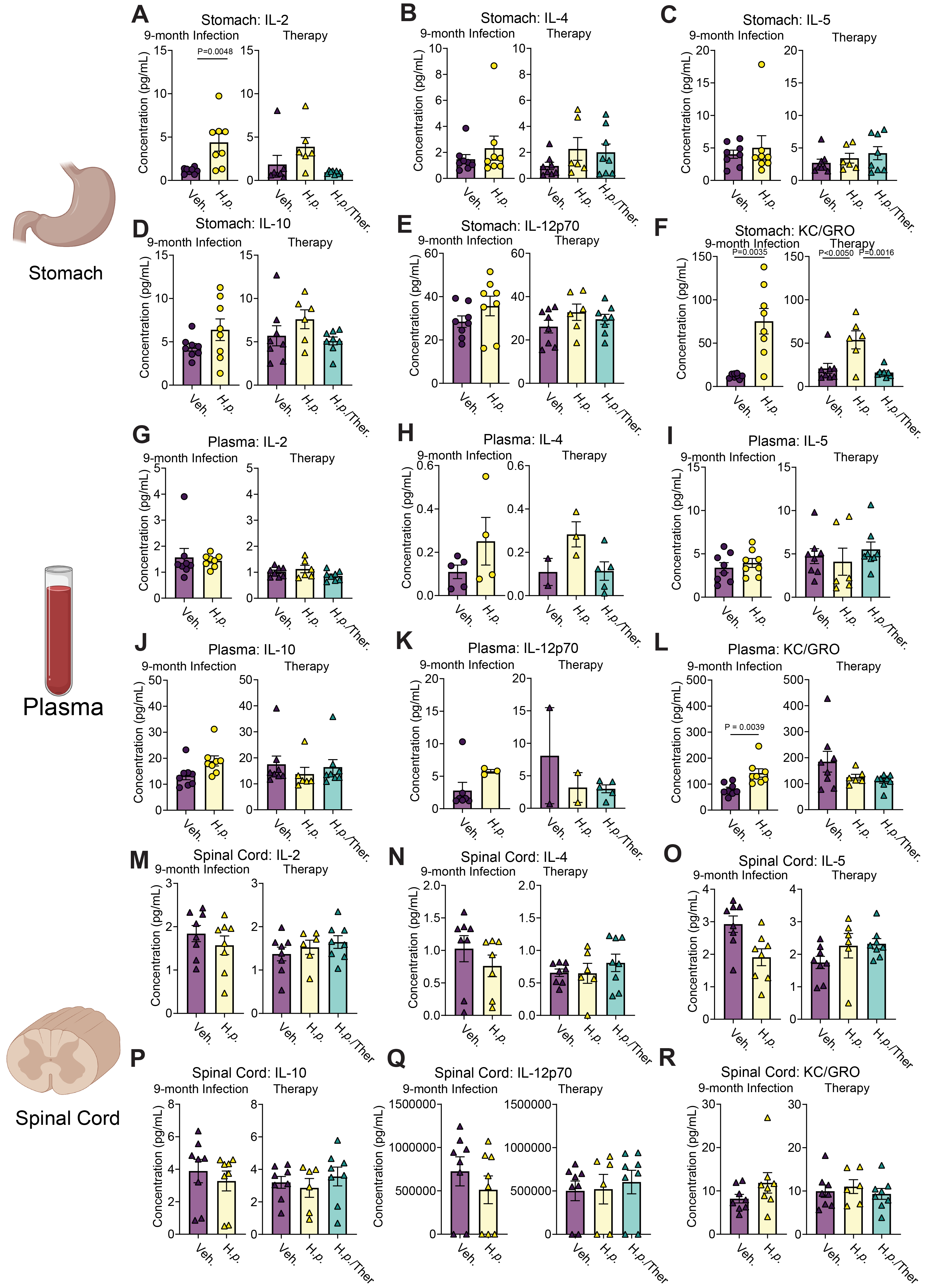
**

**Fig. S2. Further evaluation of cytokines and chemokines across tissues.** Multiplexed cytokine assay showing calculated concentration of IL-2, IL-4, IL-5, IL-10, IL-12p70, and KC/GRO levels measured in the **A-F.** stomach tissue, **G-L.** blood plasma, and **M-R.** spinal cord tissue of 9-month infection and Therapy cohort mice. For the 9-month infection cohort, n = 8 (Veh.) or n = 8 (H.p.), Welch’s T-tests were performed for **A, C, D, F, G, K,** and **R**. Unpaired T-tests were performed for the remaining panels. For the Therapy cohort, n = 8 (Veh.), 6 (*H.p.)*, or 8 (*H.p.*/Ther.), one-way ANOVA with Tukey’s multiple comparisons test was performed. Individual mice with cytokine levels below assay detection limits were excluded from analysis. Statistical significance (p < 0.05) is indicated above relevant comparisons; all other differences are non-significant.

**
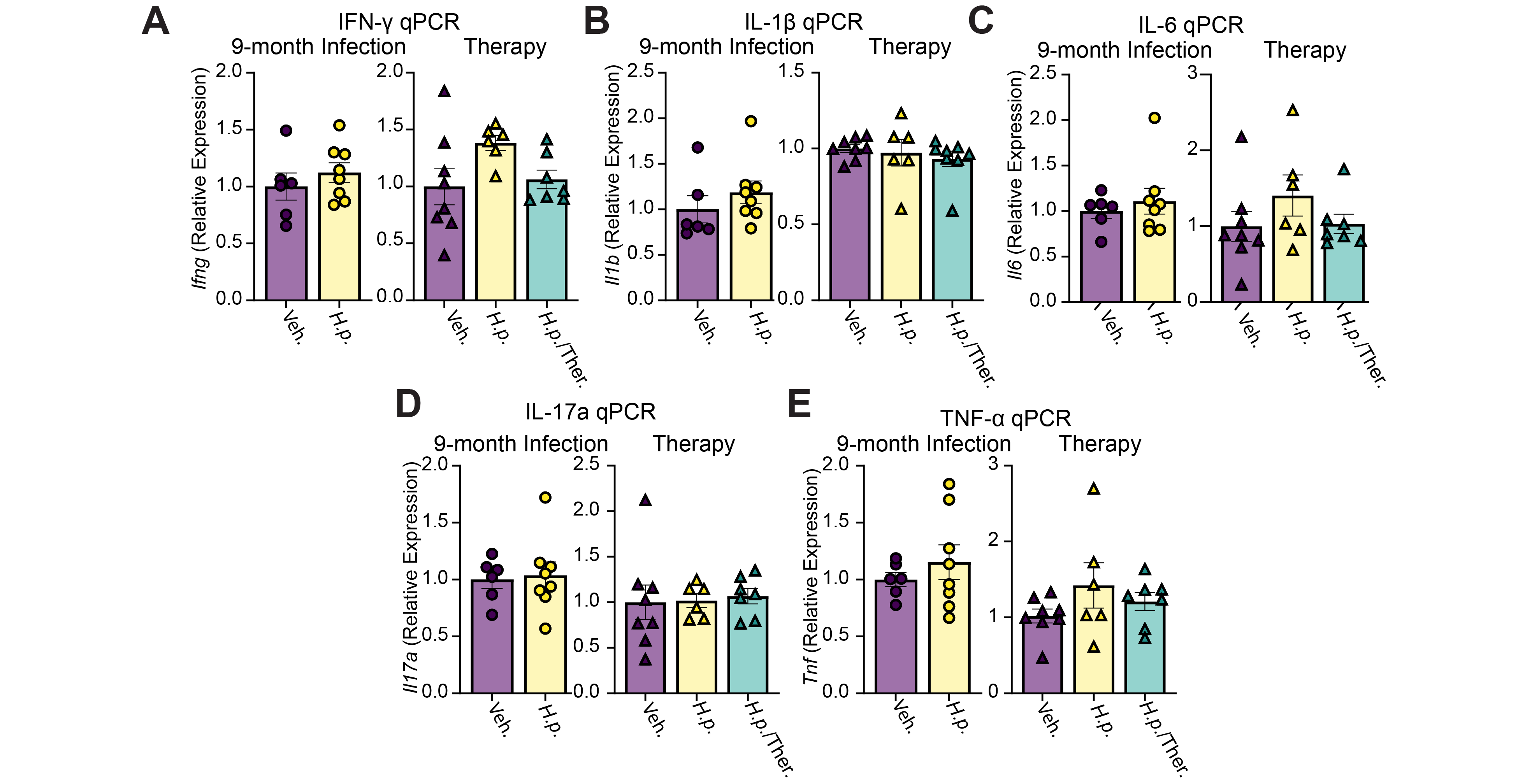
**

**Fig. S3.** **mRNA expression of proinflammatory cytokines is not significantly elevated in mouse spinal cords.** Relative expression of **A.** *Ifng*, ***B.*** *Il1b,* ***C.*** *Il6*, **D.** *Il17a*, and **E.** *Tnfa* mRNA in 9-month Infection and Therapy cohort spinal cords. n=6 (Veh.) or 8 (*H.p.)*. Welch’s T-test was performed for **E.** Unpaired T-test was performed for **A, B, C,** and **D.** For the Therapy mice, n= 8(Veh.), 6 (*H.p.),* or 7 (*H.p.*/Ther.*).*  One-way ANOVA with Tukey’s multiple comparisons test was performed. Statistical significance (p < 0.05) is indicated above relevant comparisons; all other differences are non-significant.


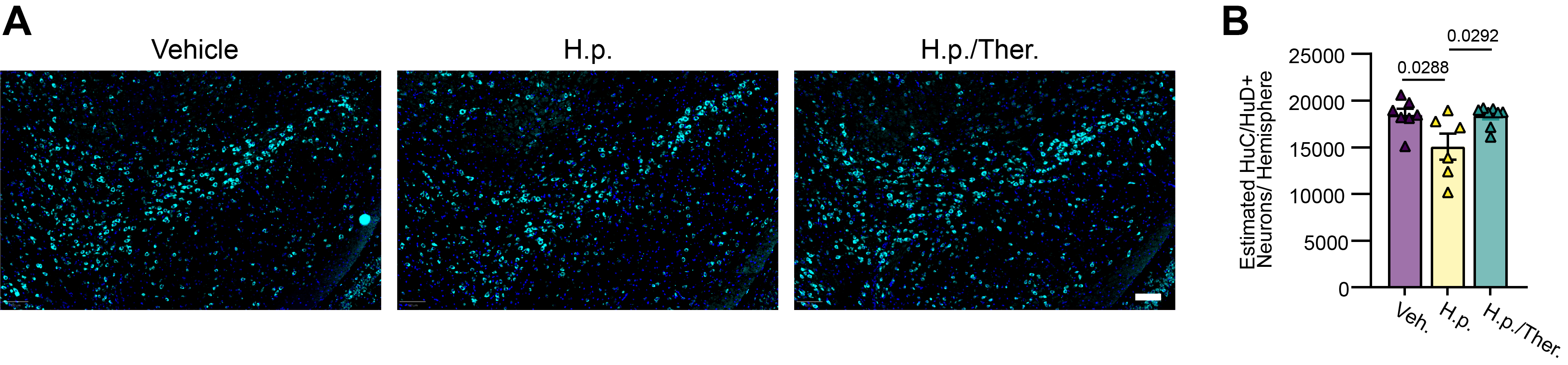


**Fig. S4. *H. pylori* infection leads to mild neurodegeneration in the substantia nigra. A.** Representative images of Therapy cohort substantia nigra stained for HuC/HuD. Scale bar = 100 µm. **B.** Estimated quantification of HuC/HuD+ cells throughout the substantia nigra shows a mild loss of neurons overall in this region. n = 7 (Veh.), 6 (*H.p.)*, or 8 (*H.p.*/Ther.), one-way ANOVA with Tukey’s multiple comparisons tests were performed. Statistical significance (p < 0.05) is indicated above relevant comparisons; all other differences are non-significant.

**
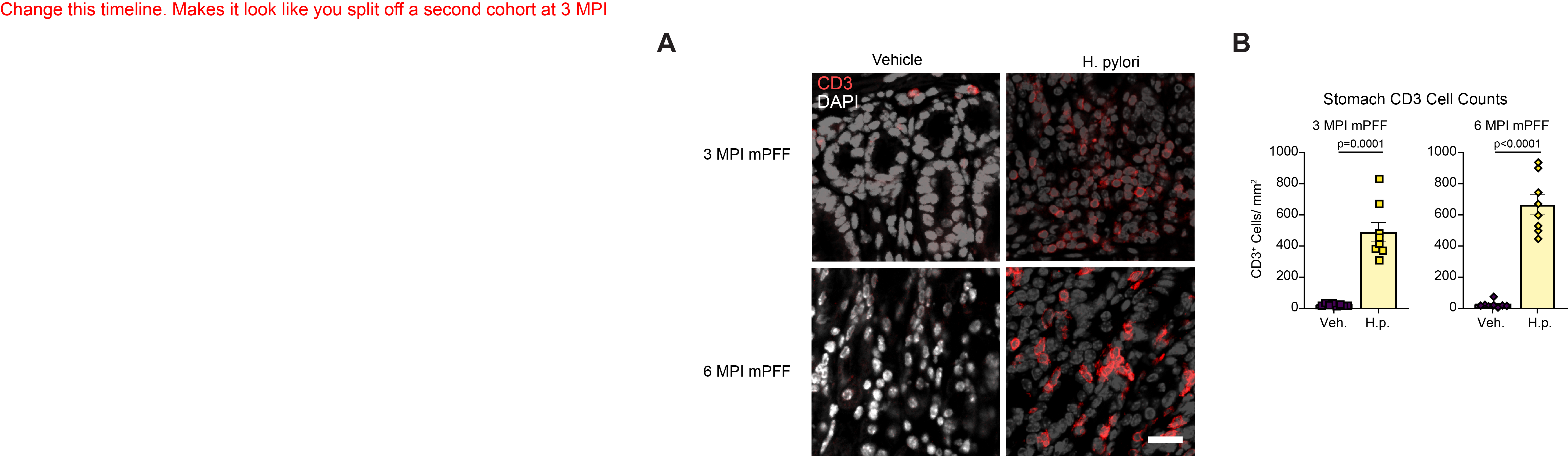
**

**Fig. S5. Dual-hit *H. pylori* model shows similar prolonged infiltration of the stomach by T-cells. A.** Representative image of 3 MPI PFF and 6 MPI PFF cohort mouse stomachs stained for CD3. Scale bar = 20 µm. **B.** Quantification of CD3^+^ cells. n= 8 (Veh.) or n=8 (*H.p.*). Welch’s T-test was performed. Statistical significance (p < 0.05) is indicated above relevant comparisons; all other differences are non-significant.

**
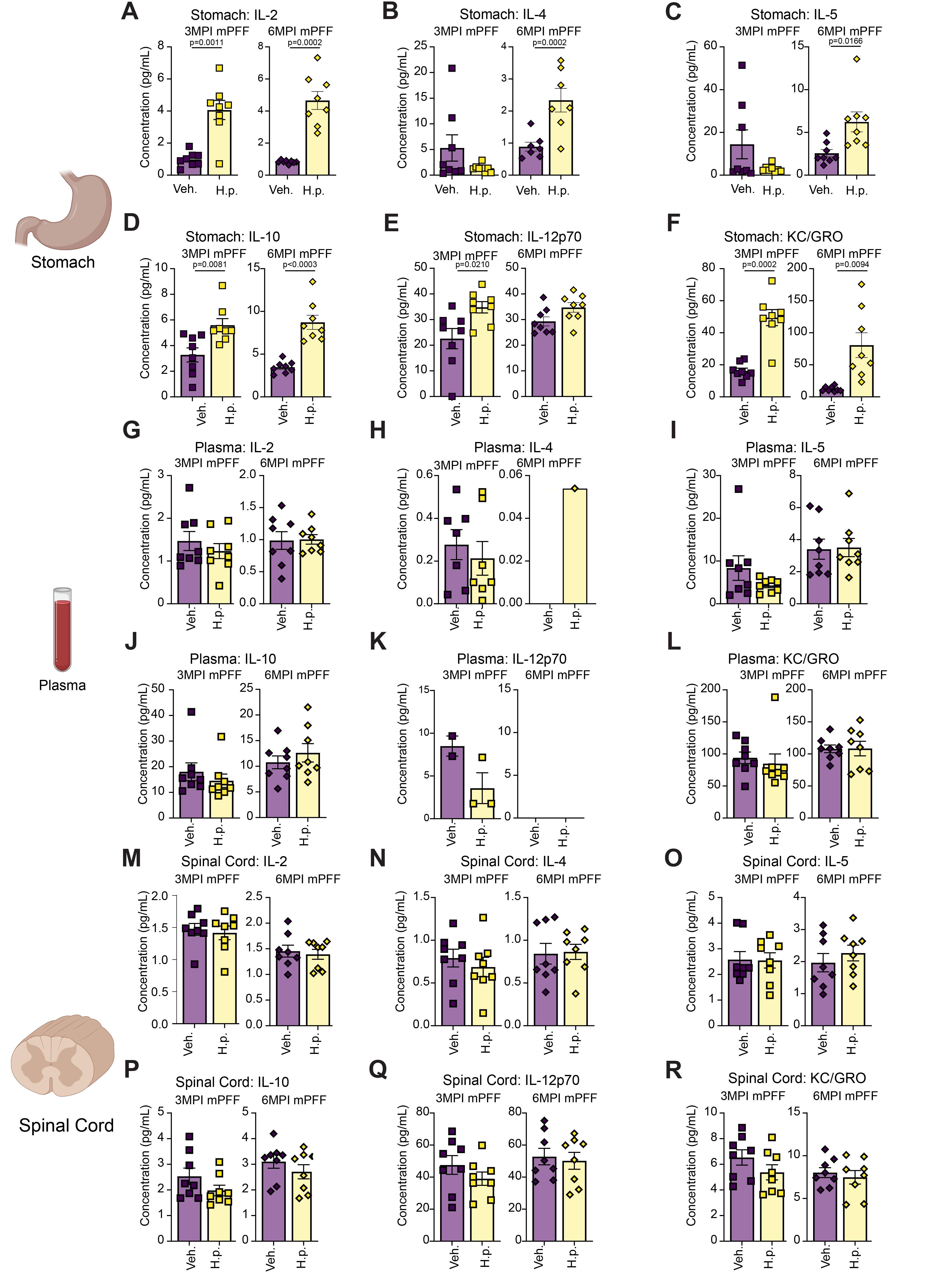
**

**Fig. S6. Further evaluation of cytokines and chemokines in α-synuclein PFF-treated mice.** Multiplexed cytokine assay showing calculated concentration of IL-2, IL-4, IL-5, IL-10, IL-12p70, and KC/GRO levels measured in **A-F.** stomach tissue, **G-L.** blood plasma, and **M-R.** spinal cord tissue of 3 MPI PFF and 6 MPI PFF cohort mice. n = 8 (Veh.) or n = 8 (H.p.), Welch’s T-tests were performed for **A, B, C, D,** and **I**. Unpaired T-tests were performed for the remaining panels. Statistical significance (p < 0.05) is indicated above relevant comparisons; all other differences are non-significant.

**
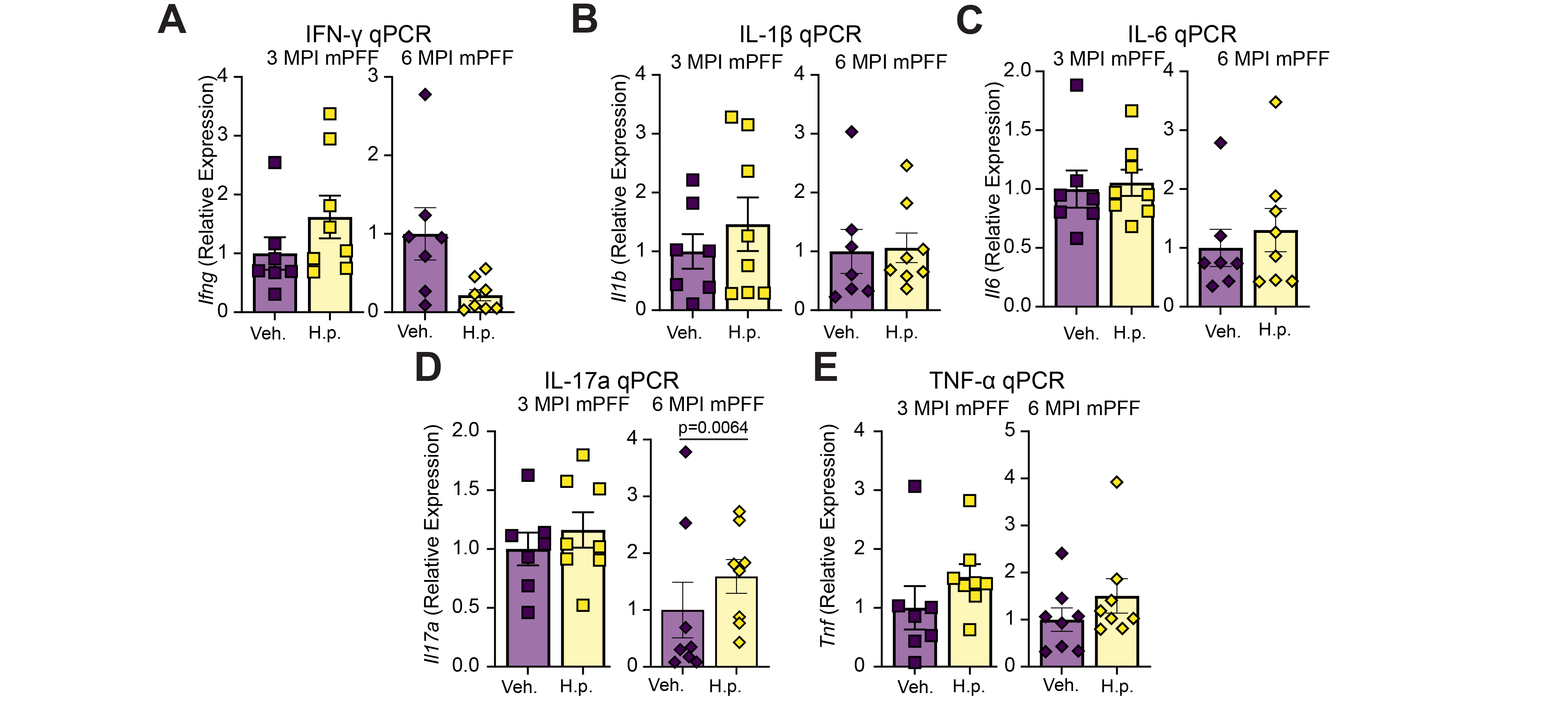
**

**Fig. S7. Addition of mPFF injections to *H. pylori* infection in mice does not alter expression of proinflammatory cytokines in spinal cords.** Relative expression of **A.** *Ifng*, ***B.*** *Il1b,* ***C.*** *Il6*, **D.** *Il17a*, and **E.** *Tnfa* mRNA in3 MPI mPFF and 6 MPI mPFF spinal cords. n=7 (Veh.) or 8 (*H.p.)*. Welch’s T-test was performed for **A** and **D.** Unpaired T-test was performed for **B, C,** and **E.** Statistical significance (p < 0.05) is indicated above relevant comparisons; all other differences are non-significant.
